# Human pluripotent stem cell process parameter optimization in a small scale suspension bioreactor

**DOI:** 10.1186/1753-6561-9-S9-O10

**Published:** 2015-12-14

**Authors:** Yonatan Y Lipsitz, Peter W Zandstra

**Affiliations:** 1 Institute of Biomaterials and Biomedical Engineering, University of Toronto, Toronto, Ontario, Canada, M5S 3E1; 2 The Donnelly Centre for Cellular and Biomolecular Research, University of Toronto, Toronto, Ontario, Canada, M5S 3E1

## Background

As cell-based therapies begin to enter clinical trials, human pluripotent stem cells (PSCs) present a reliable and robust source of cells for differentiation into all cell types in the human body[1]. PSC may one day provide cells for new treatment options for a wide range of diseases including cardiovascular disease, neurodegenerative diseases, and diabetes. The large numbers of cells required for many of these therapies necessitates the development of scalable and cost efficient technologies for manufacturing PSCs and their derivatives [2]. PSCs are an adherent cell type that is susceptible to phenotypic change in response to environmental stress. Adherent PSCs can be grown in suspension by forming aggregates of undifferentiated cells[3,4], by encapsulating PSCs in beads or hydrogels[5,6], or by adhering PSCs to the surface of microcarriers[7,8].

Most PSC suspension expansion to date has been performed in benchtop stirred-tank bioreactors operating at volumes of 50-200mL. Little bioprocess optimization has been conducted at these scales likely because of the high cost of PSC growth media and labour requirements for bioreactor growth. This study describes the use of a small-scale bioreactor system for Design-of-Experiments based PSC expansion process development. These process developments inform strategies to bring PSC-based processes into clinical production.

## Materials and methods

Human embryonic stem cells (HES2 cell line) were cultured in adherent conditions on Geltrex-coated plates. Cells were fed daily with Nutristem PSC XF Media, and were passaged with TrypLE every 5-6 days at a passage ratio of 1:24. Cells were dissociated to single cells for bioreactor seeding with TrypLE, strained through a 40μm strainer, and seeded into Nutristem media with Y-27632 ROCK inhibitor prior to bioreactor seeding. Culture media was exchanged at regular intervals by allowing aggregates to settle, removing half of the culture media, and replacing fresh media (without ROCK inhibitor).

**Figure 1:**
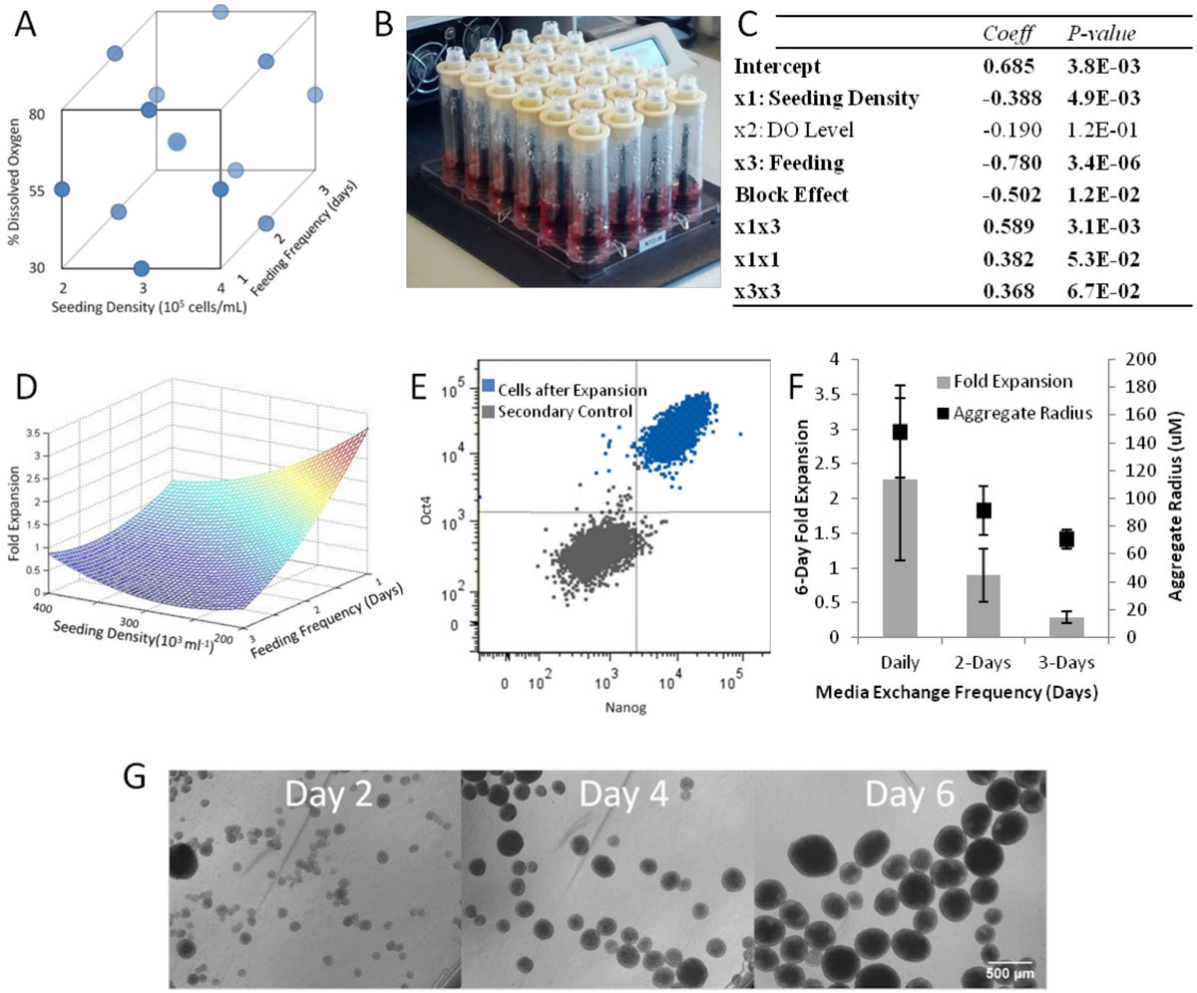
**Pluripotent stem cell bioprocess optimization in small scale suspension cultures**. (a) Box Behnken Experimental Design. This experiment used 14 wells of the cassette - 12 wells for points along the edges of the experimental design and 2 centrepoint replicates. This experimental design allows for the identification of higher-order effects while using fewer than the 24 wells available in the Micro-24 cassette. (b) Micro-24 Cassette. The PRC-Cell culture cassette with Type A caps is loaded into the Micro-24 system. (c) Significant parameters in quadratic regression of PSC expansion. Bold factors are significant, including seeding density, feeding, interaction, and quadratic factors of these two parameters. Block effect was also found to be significant, which indicates variability between passage number of the same cell line. (d) Surface response curve of significant parameters investigated in the bioreactor expansion of HES2 PSCs. Seeding density and feeding frequency were found to significantly influence the fold expansion of HES2 cells, with both quadratic and interaction effects between these two parameters also observed. (e) Representative FACS Plot of PSCs after six days of bioreactor expansion. In all conditions tested, >90% Oct4 Sox2 cell populations were observed. (f) Relationship between fold expansion and aggregate radius for cells seeded at 2*10^5 ^cells/mL. While frequent feeding yields the highest fold expansion and is correlated to larger final aggregate radius after six days of expansion, it also results in the highest variability in aggregate size. Error bars represent one standard deviation. (g) Formation and growth of PSC aggregates spontaneously in bioreactor over six days of culture.

To study the effects of feeding frequency (media exchange every 1, 2, or 3 days), dissolved oxygen level (80%, 55%, 30%), and seeding density (2x105, 3x105, 4x105cells/mL) on PSC expansion, a three level, three factor Box-Behnken experimental design was developed including three centrepoint replicates (Figure 1a). A Micro-24 Bioreactor System (Pall) was used with PRC-Cell Culture cassettes (Figure 1b) and operated with 2mL volume at 7.3 pH, 37°C, and 400rpm. A quadratic regression model identified the significance of the three factors, their quadratic effects, and their interaction effects. Three representative images were captured of cell aggregates, and aggregate size was determined using the aggregate size tool in the ImageJ software. Cells were then dissociated as described above and counted by Trypan Blue exclusion. Dissociated cells were fixed and Oct4 and Nanog levels quantified by flow cytometry.

## Results

Preliminary results indicated that ROCK inhibitor is essential for cell survival and expansion (data not shown). Cell expansion was strongly dependent on feeding frequency (Figure 1c): daily cell feeding resulted in significantly higher cell densities. Seeding density also significantly influenced cell expansion (Figure 1c) with both higher and lower densities producing higher cell expansion than intermediate densities. The optimal cell density found in this experiment, 2x105cells/mL, was an endpoint within the experimental conditions tested; however, when testing a lower cell density (1x105 cells/mL), cells formed large aggregates with low cell viability (data not shown). Fold-increase in cell numbers was plotted against the significant input parameters (Figure 1d) to depict the surface response in this experiment. The curvature of the surface response is characteristic of the quadratic parameters that were significant in the regression of the experimental data. Significant interaction effects were observed between seeding density and feeding frequency (Figure 1c).

The final composition of PSCs in the bioreactor-expanded cells was quantified by flow cytometry. All conditions tested retained >90% Oct4+ Nanog+ pluripotent PSCs (representative plot in Figure 1e) after six days of bioreactor culture.

PSC were observed to spontaneously form uniform aggregates when seeded into the bioreactor as single cells, which expanded to uniform PSCs aggregates at 4 and 6 days of expansion (Figure 1f and representative images in Figure 1g). Aggregate size was found to also be a function of feeding frequency (Figure 1f), and was correlated with final fold increase in cell number. Frequent feeding resulted in increased variability in aggregate size (Figure 1f).

## Discussion and conclusions

The process optimum found in this study raises several interesting questions for future experimentation. The most significant factor was feeding frequency, with more frequent feeding increasing yields; however, it is unknown what drives the dramatic effects of media supplementation on the resultant increased yields. If growth factors are being depleted, repeating these experiments with culture media that is more enriched in the necessary growth factors could generate different trends than those observed here. Similarly, if secreted factors or metabolites impede cell growth, modified media exchange strategies may be appropriate.

While the initial experimental design investigated the effects of oxygen tension on cell production, this parameter was found to be insignificant in these studies. Previous literature indicates that hypoxic conditions are beneficial for PSC growth [9], as well as the expansion of various other stem cell types[10,11]. In our experiment, cells were not previously adapted to hypoxic conditions, which may impact cell responses during oxygen dependence transitions and limited the observed significance of this parameter. Alternatively, the dissolved oxygen setpoint in the hypoxic condition may have been too high to observe beneficial growth due to differences in mass transfer between the Micro-24 system used and systems used by others.

For future experimentation, the effects of increasing culture volume and the resulting effects on the shear stress that cells are exposed to should be explored. Not only will this increase understanding of bioprocess parameters in this platform, but increasing cell volume above the 2mL point, which is the low end of the operating range of the device, could decrease experimental variability.

The Micro-24 system represents a tool useful for relating media components, cellular parameters, and bioprocess parameters to cell manufacturing in the cell therapy industry. The results of this experiment can guide the development of process design spaces for PSC expansion processes.

## References

[B1] Thomson JA Itskovitz-Eldor J Shapiro SS Waknitz MA Swiergiel JJ Marshall VS Jones JM Embryonic stem cell lines derived from human blastocysts Science 1998 282 5391 1145 1147 9804556 10.1126/science.282.5391.1145

[B2] Kirouac DC Zandstra PW The Systematic Production of Cells for Cell Therapies Cell stem cell 2008 3 4 369 381 18940729 10.1016/j.stem.2008.09.001

[B3] Amit M Laevsky I Miropolsky Y Shariki K Peri M Itskovitz-Eldor J Dynamic suspension culture for scalable expansion of undifferentiated human pluripotent stem cells Nat Protoc 2011 6 5 572 579 21527915 10.1038/nprot.2011.325

[B4] Zweigerdt R Olmer R Singh H Haverich A Martin U Scalable expansion of human pluripotent stem cells in suspension culture Nat Protoc 2011 6 5 689 700 21527925 10.1038/nprot.2011.318

[B5] Siti-Ismail N Bishop AE Polak JM Mantalaris A The benefit of human embryonic stem cell encapsulation for prolonged feeder-free maintenance Biomaterials 2008 29 29 3946 3952 18639332 10.1016/j.biomaterials.2008.04.027

[B6] Dang SM Gerecht-Nir S Chen J Itskovitz-Eldor J Zandstra PW Controlled, scalable embryonic stem cell differentiation culture Stem Cells 2004 22 3 275 282 15153605 10.1634/stemcells.22-3-275

[B7] Oh SK Chen AK Mok Y Chen X Lim UM Chin A Choo AB Reuveny S Long-term microcarrier suspension cultures of human embryonic stem cells Stem Cell Res 2009 2 3 219 230 19393590 10.1016/j.scr.2009.02.005

[B8] Storm MP Orchard CB Bone HK Chaudhuri JB Welham MJ Three-dimensional culture systems for the expansion of pluripotent embryonic stem cells Biotechnol Bioeng 2010 107 4 683 695 20589846 10.1002/bit.22850 PMC3580883

[B9] Niebruegge S Bauwens CL Peerani R Thavandiran N Masse S Sevaptisidis E Nanthakumar K Woodhouse K Husain M Kumacheva E Generation of Human Embryonic Stem Cell-Derived Mesoderm and Cardiac Cells Using Size-Specified Aggregates in an Oxygen-Controlled Bioreactor Biotechnology and Bioengineering 2009 102 2 493 507 18767184 10.1002/bit.22065

[B10] Roy S Tripathy M Mathur N Jain A Mukhopadhyay A Hypoxia improves expansion potential of human cord blood-derived hematopoietic stem cells and marrow repopulation efficiency European journal of haematology 2012 88 5 396 405 22268587 10.1111/j.1600-0609.2012.01759.x

[B11] Tsai CC Chen YJ Yew TL Chen LL Wang JY Chiu CH Hung SC Hypoxia inhibits senescence and maintains mesenchymal stem cell properties through down-regulation of E2A-p21 by HIF-TWIST Blood 2011 117 2 459 469 20952688 10.1182/blood-2010-05-287508

